# The intervention of curcumin on rodent models of hepatic fibrosis: A systematic review and meta-analysis

**DOI:** 10.1371/journal.pone.0304176

**Published:** 2024-05-23

**Authors:** Yun-Hang Chu, Bing-Yao Pang, Ming Yang, Qi Meng, Yan Leng

**Affiliations:** 1 College of Traditional Chinese Medicine, Changchun University of Traditional Chinese Medicine, Changchun, China; 2 Department of Hepatology, The Affiliated Hospital of Changchun University of Traditional Chinese Medicine, Changchun, China; Helwan University, EGYPT

## Abstract

**Objective:**

This study aimed to evaluate the intervention effect of curcumin on hepatic fibrosis in rodent models through systematic review and meta-analysis, in order to provide meaningful guidance for clinical practice.

**Methods:**

A systematic retrieval of relevant studies on curcumin intervention in rats or mice hepatic fibrosis models was conducted, and the data were extracted. The outcome indicators included liver cell structure and function related indicators, such as alanine aminotransferase (ALT), aspartate aminotransferase (AST), alkaline phosphatase (ALP), albumin (ALB), ratio of albumin to globulin (A/G), total bilirubin (TBIL), bax protein, bcl-2 protein and index of liver, as well as the relevant indicators for evaluating the degree of hepatic fibrosis, such as hyaluronic acid (HA), laminin (LN), type I collagen (Collagen I), type III collagen (Collagen III), type III procollagen (PCIII), type III procollagen amino terminal peptide (PIIINP), type IV collagen (IV-C), interleukin-6 (IL-6), tumor necrosis factor-α (TNF-α), α-Smooth muscle actin (α-SMA), hydroxyproline (HYP), platelet derived factor-BB (PDGF-BB), connective tissue growth factor (CTGF) and transforming growth factor-β1 (TGF-β1), and oxidative stress-related indicators, such as superoxide dismutase (SOD), malondialdehyde (MDA) and glutathione peroxidase (GSH-Px). These results were then analyzed by meta-analysis. Studies were evaluated for methodological quality using the syrcle’s bias risk tool.

**Results:**

A total of 59 studies were included in the meta-analysis, and the results showed that curcumin can reduce the levels of ALT, AST, ALP, TBIL, bax protein, and index of liver in hepatic fibrosis models. It can also reduce HA, LN, Collagen I, Collagen III, PCIII, PIIINP, IV-C, TNF-α, α-SMA, HYP, PDGF-BB, CTGF, TGF-β1 and MDA, and increase the levels of ALB, A/G, SOD, and GSH-Px in the hepatic fibrosis models. However, the effects of curcumin on bcl-2 protein, IL-6 in hepatic fibrosis models and index of liver in mice were not statistically significant.

**Conclusion:**

The analysis results indicate that curcumin can reduce liver cell apoptosis by maintaining the stability of liver cell membrane, inhibit the activation and proliferation of hepatic stellate cells by reducing inflammatory response, and alleviate tissue peroxidation damage by clearing oxygen free radicals.

## Introduction

Hepatic fibrosis is a pathological process caused by various pathogenic factors such as hepatitis virus, ethanol, drugs and toxins, parasites, metabolism and genetics, bile stasis, immune abnormalities, etc., which lead to liver cell damage and immune inflammation activation [1]. This activation subsequently stimulates hepatic stellate cells (HSC) and results in excessive deposition of extracellular matrix (ECM) within the liver, leading to liver structural and functional damage. While hepatic fibrosis represents a liver repair response, the persistent accumulation of ECM can harm the tissue structure, causing blood flow disorders. This can lead to the progression of hepatic fibrosis into cirrhosis, even liver cancer, ultimately resulting in liver failure or death. At present, there is no reliable data on the incidence rate and prevalence of liver fibrosis, but according to the epidemiological studies and clinical pathological evolution of various chronic liver diseases, it can be inferred that liver fibrosis is quite common in the population: more than 1.5 billion people worldwide suffer from chronic liver disease, and the most common causes are in turn nonalcoholic fatty liver disease, hepatitis B virus (HBV), hepatitis C virus and alcoholic liver disease [2]. The resulting cirrhosis accounts for 1.82% of the global disease burden, resulting in 1.2 million deaths annually [3]. Hepatocellular carcinoma (HCC), as the second leading cause of cancer death worldwide, accounts for 42%, 18%, and 6% of the incidence of HBV, alcoholic hepatitis, and non-alcoholic steatohepatitis, respectively [4]. More than 80% of HCC occurs in fibrotic or sclerotic liver, leading to over 790000 deaths annually [5]. It is now believed that there is still a possibility of reversing hepatic fibrosis, and controlling the development of hepatic fibrosis is the key to blocking the occurrence of cirrhosis and liver cancer [6].

Curcumin is a natural active ingredient mainly derived from the tubers of *Curcuma aromatica Salisb*., *C*.*longa L*., *C*.*zedoaria (Berg*.*) Rosc*., and *Acorus calamus L*. in the Araceae family [7]. Its diverse pharmacological activities are associated with its antioxidant and anti-inflammatory properties [8]. In the realm of hepatic fibrosis, numerous studies have been conducted by scholars in recent years on the effects and molecular mechanisms of curcumin in animal models [9]. To elucidate the impact of curcumin on hepatic fibrosis, this study performed a meta-analysis to assess the therapeutic effect of curcumin on this condition. The aim is to validate the efficacy of curcumin in treating hepatic fibrosis, thereby providing valuable insights for its clinical application.

## Materials and methods

### Protocol and registratsion

This systematic review has been registered at the PROSPERO International System Review Prospective Registry (registration number: CRD42023486432) to help avoid duplication and reduce opportunities for reporting bias by comparing completed evaluations with program plans. We followed the preferred reporting guidelines of the Preferred Reporting Item for Systematic Reviews and Meta-Analysis Statement (PRISMA) to ensure the completeness and accuracy of the research.

### Literature search

Through databases such as PubMed, Embase, Cochrane Central Controlled Trial Registry, Web of Science, China National Knowledge Infrastructure (CNKI), China Biomedical Literatsure Database (CBM), China Science and Technology Journal Database (VIP), and Wanfang Database. The time range is from database establishment to November 10, 2023. The search keywords include the following three parts: 1) curcumin, 2) hepatic fibrosis or liver fibrosis, 3) rat or mice. The three keywords are connected by "and".

The inclusion criteria for the article are as follows:

1. Research subjects: hepatic fibrosis models in rats or mice. The preparations method of the model must be recognized.
2. Intervention measures: The medication used must be curcumin or curcumin preparations. The experimental group cannot use other drugs that may affect the liver separatsely. The control group did not use any medication or only used placebo.
3. Result indicators: The indicators for evaluating drug efficacy must be presented in numerical form to ensure that key indicators, including their mean and standard deviation, can be directly extracted or indirectly calculated.

The exclusion criteria for the article are as follows:

1. The article is a meta-analysis, systematic review, or incomplete communication information.
2. The study involves experimental animals other than rats and mice.
3. *Ex vivo*, *in vitro* and in silicon models.
4. The experimental group or control group involves the use of other drugs.
5. Report on results unrelated to the effect of hepatic injury in rodents with hepatic fibrosis.
6. The data of evaluation indicators is incomplete (such as the measurement units of unmarked evaluation indicators).

The following information were extracted from qualified studies: author, year of publication, drugs used, modeling method, number of models, mode of administratsion, dosage, detection indexes, detection index unit, and detection results [10–68]. Finally, a summary of the outcome indicators was provided. When there were fewer than three articles on outcome indicators, they were excluded from the study. To evaluate the quality of the included studies, we used the Systemic Review Center for Laboratsory Animal Experimentation bias risk tool (syrcle’s bias risk tool) for animal studies [69, 70]. Syrle’s bias risk tool is based on the Cochrane bias risk tool and adjusted for specific aspects of bias that play a role in animal intervention studies. In terms of data extraction, two researchers independently conducted a preliminary screening of literature titles and abstracts. After the initial screening, the literature was re screened by reading the entire text; Then cross check the screening results, while considering the qualification criteria and recording the selection and exclusion reasons for each step. If there is any disagreement, it can be decided whether to include it through joint discussion, and if necessary, a third researcher can assist in resolving it. If the information in the article is incomplete or unclear, the original research author will be contacted and the information will be obtained through email consultation.

## Statistical methods

Perform meta-analysis using RevMan 5.1 software.Quantitative data were expressed as mean ± standard deviation ($\bar{X}$±SD). Use Cochran’s Q test and I^2^ test to evaluate the existence and severity of heterogeneity. When both p < 0.1 and I^2^ > 50%, it was considered that there was heterogeneity, and the random-effects model was used for meta-analysis. Otherwise, the fixed-effects model was used. In Stata 12.0, Begg rank correlation method and Egger linear regression method were used to evaluate publication bias, and no significant publication bias was considered when P>0.05.

## Result

### Results of the literatsure review

**Fig 1:**
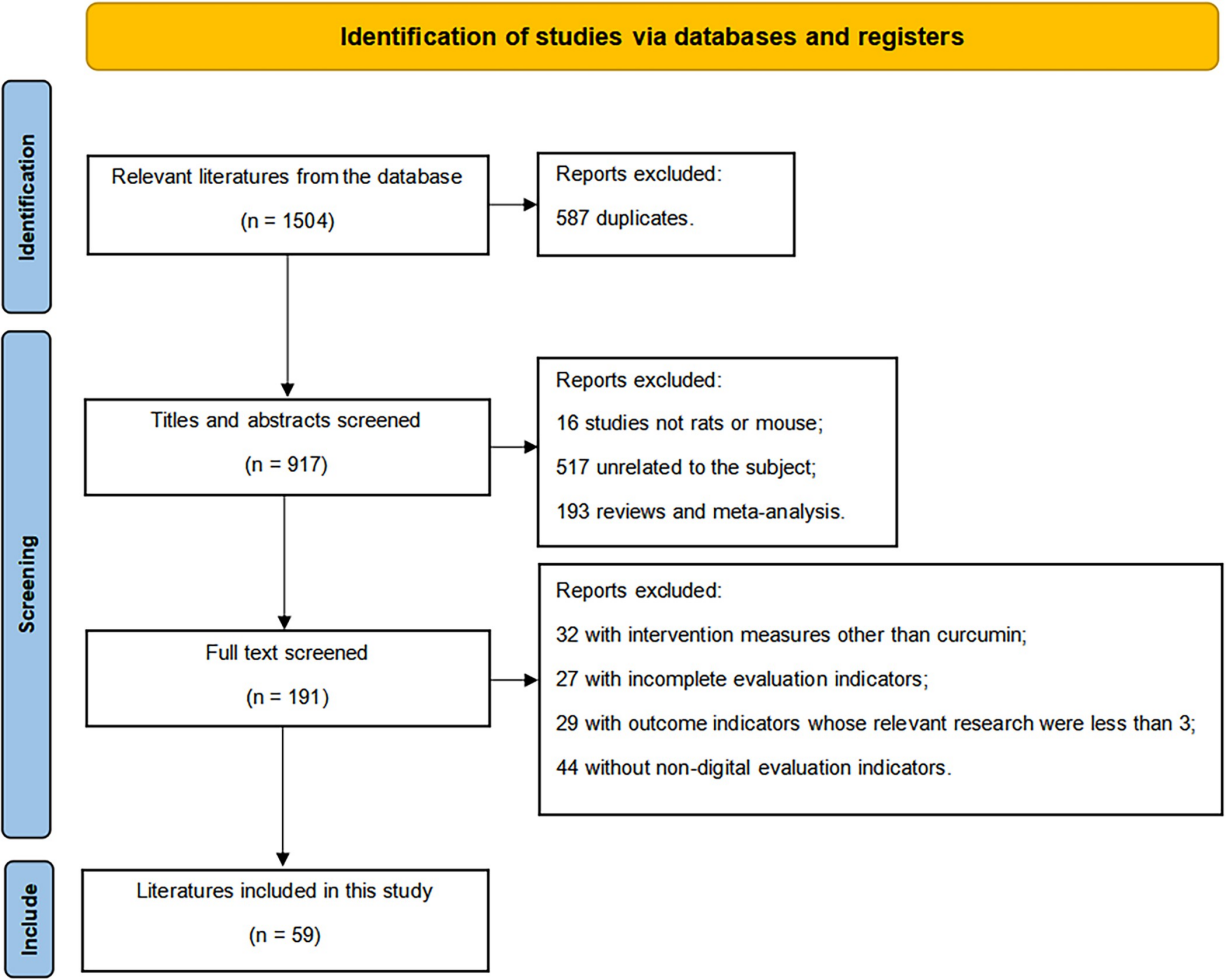
PRISMA flow chart illustrating steps and details of the identification and selection process for the systematic review.

The file retrieval process diagram is shown in Fig 1. As shown in Fig 1, a total of 1504 studies were retrieved from the databases. Of these, 587 were repeated studies that were then excluded, and 917 studies remained. Among the 917 articles, 16 articles studied animals that were not rats or mice, 517 articles were unrelated to the research topic, 193 articles were reviews or meta-analyses, 32 studies involved intervention methods other than curcumin, 27 studies had incomplete data, there were 29 studies that involved outcome indicators whose relevant research were less than 3, and 44 studies had evaluation indicators that were not numerical. Finally, 59 articles were included in the meta-analysis. All included literatures contain the control group,model group, and curcumin treatment group. The study ID, experimental subjects, modeling methods, and other research features were extracted, as shown in Table 1.

**Table 1:** Study ID, study subject, modeling method and other research characteristics of included literatures.

Serial number	Study ID	Study subjects (model group/ curcumin group)	Modeling method	Administration method	Dosage (mg/kg)	Outcome indexes
1	CWS2013 [10]	Rats (7/7)	Subcutaneous injection of CCL4	Gavage	200	HA, IV-C, TGF-β1(blood serum)
2	CWL2013 [11]	Rats (10/10)	DMN was injected intraperitoneally	Gavage	200	ALT, AST, PC-Ⅲ, IV-C, HA, TGF-β1(liver tissue)
3	DSE2011 [12]	Rats (10/10)	Transdermal infection of Schistosoma cercariae	Gavage	300	ALT, AST, TBIL, ALB, HYP
4	DYH2012 [13]	Rats (15/15)	Subcutaneous injection of CCL4	Tail vein injection	100	ALT, AST, HYP
5	FV2010 [14]	Mice (6/6)	MCD diet	Intraperitoneal injection	25	Index of liver
6	GCS2009 [15]	Rats (12/12)	CCL4 was injected intraperitoneally	Gavage	200	α-SMA
7	HH2009 [17]	Rats (8/9)	Subcutaneous injection of CCL4	Gavage	400	A/G, TGF-β1(liver tissue)
8	HHY2008 [18]	Rats (8/9)	Subcutaneous injection of CCL4	Gavage	400	ALT, AST, ALP, CollagenⅠ
9	HHY2009 [16]	Rats (9/8)	Subcutaneous injection of CCL4	Gavage	400	ALT, AST, ALP, A/G, LN, PⅢNP, IV-C, CollagenⅠ, HA
10	HJX2009 [19]	Rats (10/10)	Subcutaneous injection of CCL4	Gavage	200	HA, LN, PCⅢ, IV-C
11	HLM2019 [20]	Mice (7/10)	Intraperitoneal TAA	Gavage	200	ALT, AST, TBIL, HA, LN, PCⅢ, IV-C, HYP
12	HN2007 [21]	Rats (10/10)	CCL4 was injected intraperitoneally	Gavage	200	HA, LN, IV-C
13	HSM2006 [22]	Rats (5/5)	CCL4 was injected intraperitoneally	Gavage	300	HA, TGF-β1(blood serum), α-SMA, PDGF-BB, TGF-β1(liver tissue)
14	HTH2009 [23]	Rats (11/15)	Subcutaneous injection of CCL4	Gavage	100	HYP
15	HTH2008 [24]	Rats (11/15)	Subcutaneous injection of CCL4	Gavage	100	bcl-2 proten, bax proten
16	HTHh2008 [25]	Rats (15/15)	Subcutaneous injection of CCL4	Gavage	100	α-SMA
17	HXH2015 [26]	Rats (10/10)	Subcutaneous injection of CCL4	Tail vein injection	400	ALT, AST
18	HXHh2015 [27]	Rats (15/15)	Subcutaneous injection of CCL4	Tail vein injection	400	ALT, AST
19	JH2010 [28]	Rats (8/10)	Subcutaneous injection of CCL4	Gavage	400	ALT, AST, Collagen I, Collagen Ⅲ
20	JWW2009 [29]	Mice (10/10)	CCL4 was injected intraperitoneally	Gavage	50	ALT, α-SMA, CTGF, HA
21	LHY2007 [30]	Mice (10/10)	CCL4 was administered intragastrically	Gavage	200	Index of liver, ALT, AST, HA, IV-C, TGF-β1(blood serum)
22	LJL2010 [31]	Rats (8/10)	Subcutaneous injection of CCL4	Gavage	200	ALT, AST, HA, LN, PCⅢ, IV-C, CollagenⅢ, CollagenI
23	LYG2002 [33]	Rats (7/8)	Subcutaneous injection of CCL4	Gavage	200	ALT, AST, HA, LN
24	LYG2005 [34]	Rats (8/8)	DMN was injected intraperitoneally	Gavage	200	ALT, ALP, A/G, SOD, GSH-Px, HA, LN
25	LYGg2002 [32]	Mice (10/10)	CCL4 was injected intraperitoneally/ D-GaLN intraperitoneally	Gavage	200	ALT, AST
26	LZX2019 [35]	Rats (10/10)	Intraperitoneal TAA	Gavage	400	ALT, AST, HA, LN, PCⅢ
27	MAMAMA2012 [36]	Rats (10/10)	CCL4 was injected intraperitoneally	Gavage	200	ALT, AST, TBIL
28	MEW2012 [37]	Mice (8/8)	Intraperitoneal TAA	Gavage	200	ALT, AST, Index of liver
29	MJ2011 [38]	Rats (12/11)	CCL4 was injected intraperitoneally	Gavage	200	Index of liver, ALT, AST, ALP, HA, LN, PCⅢ
30	MMA2016 [39]	Mice (12/12)	CCL4 was injected intraperitoneally	Gavage	200	ALT, AST, ALP, ALB, MDA, SOD, GSH-Px
31	OYP2016 [40]	Rats (10/10)	CCL4 was injected intraperitoneally	Gavage	40	α-SMA
32	PCW2014 [41]	Rats (10/10)	DMN was injected intraperitoneally	Gavage	50	ALT, AST
33	RLJ2019 [42]	Rats (10/10)	CCL4 was injected intraperitoneally	Gavage	200	ALT, AST, ALB
34	RXF2011 [43]	Rats (12/12)	DMN was injected intraperitoneally	Gavage	50	ALT, AST, SOD, GSH-Px, MDA
35	SCJ2020 [44]	Mice (8/8)	CCL4 was injected intraperitoneally	Gavage	400	ALT, AST, IL-6, TGF-β1(liver tissue), Collagen I, α-SMA, TNF-α
36	SESE2020 [45]	Rats (8/8)	Bile duct ligation	Gavage	100	ALT, AST, TBIL, α-SMA, TGF-β1(liver tissue), Collagen I, MDA
37	SJ2006 [46]	Rats (10/12)	Subcutaneous injection of CCL4	Gavage	400	HA, LN, PCIII, IV-C
38	SJCc2008 [47]	Rats (8/10)	CCL4 was injected intraperitoneally	Gavage	400	ALT, AST, HA, LN, PC-Ⅲ
39	SJC2008 [48]	Rats (12/12)	CCL4 was injected intraperitoneally	Gavage	200	CTGF
40	SJC2007 [49]	Rats (12/12)	CCL4 was injected intraperitoneally	Gavage	200	MDA, PDGF, TGF-β1(liver tissue)
41	SN2012 [50]	Rats (15/15)	Subcutaneous injection of CCL4	Tail vein injection	400	ALT, AST, HYP, HA, LN, PCIII
42	SSC2007 [51]	Rats (10/12)	Subcutaneous injection of CCL4	Gavage	400	TGF-β1(liver tissue)
43	TFL2012 [52]	Rats (9/8)	CCL4 was injected intraperitoneally	Gavage	150	Index of liver, AST, ALT
44	WJ2015 [53]	Rats (7/7)	Bile duct ligation	Gavage	400	ALT, AST, TBIL
45	WLF2011 [54]	Rats (10/12)	Subcutaneous injection of CCL4	Gavage	400	bcl-2 proten, bax proten
46	WRR2019 [55]	Rats (10/10)	CCL4 was injected intraperitoneally	Gavage	100	ALT, AST, LN, PIIINP, HA, IV-C
47	WTH2021 [56]	Mice (10/10)	High fat diet	Gavage	300	ALT, AST, IL-6, TNF-α
48	WXJ2015 [57]	Rats (10/10)	CCL4 was injected intraperitoneally	Gavage	200	MDA, TGF-β1(liver tissue), PDGF, CTGF
49	WZK2020 [58]	Rats (8/8)	CCL4 was injected intraperitoneally	Gavage	5	ALT, AST, Index of liver
50	XZY2018 [59]	Mice (3/3)	CCL4 was injected intraperitoneally	Gavage	100	ALT, AST, TNF-α, IL-6, SOD, MDA, GSH-Px
51	YGR2007 [60]	Rats (8/10)	CCL4 was injected intraperitoneally	Gavage	400	TGF-β1(blood serum), TNF-α, SOD, MDA, HYP, Collagen I, Collagen Ⅲ, PDGF, α-SMA
52	YWFf2003 [61]	Rats (5/7)	DMN was injected intraperitoneally	Gavage	100	Index of liver, ALT, ALP, A/G, SOD, GSH-PX, HA, LN, HYP
53	ZZD2009 [62]	Rats (8/8)	DMN was injected intraperitoneally	Gavage	200	ALT, AST, TP, ALB, A/G, HA, LN, PCIII, TNF-α, SOD, MDA, HYP
54	ZY2015 [63]	Rats (8/8)	Subcutaneous injection of CCL4	Gavage	200	SOD, MDA
55	ZXR2007 [64]	Rats (12/10)	CCL4 was injected intraperitoneally	Gavage	100	Collagen I, Collagen Ⅲ
56	ZXH2010 [65]	Mice (7/8)	CCL4 was injected intraperitoneally	Gavage	100	ALT, AST, bcl-2 proten, bax proten
57	ZCB2020 [66]	Mice (12/12)	CCL4 was injected intraperitoneally	Gavage	200	HA, LN, IV-C, PⅢP
58	YZ2014 [67]	Rats (10/10)	CCL4 was injected intraperitoneally	Gavage	1200	ALT, AST, ALP, ALB, TP
59	YYR2022 [68]	Rats (10/10)	A high-fat, high-sugar diet	Gavage	200	Index of liver

We evaluated the quality of the included literature using the experimental animal experimental system evaluation center (SYRCLE) bias risk (RoB) tool developed based on the Cochrane randomized controlled trial bias risk assessment tool, in order to minimize the differences in bias risk assessment in animal intervention experimental system evaluations. As shown in Table 2. 52.5% (n = 31) described the method of random housing [12, 14, 19–22, 24, 29, 36–40, 42, 44–46, 48, 49, 51–53, 55, 56, 58, 60, 63–65, 67, 68]. 13.6% (n = 8) described the random sequence generation method, such as fully randomized designs, simple random sampling methods, and random block methods [11, 21–24, 29, 41, 44]. 1.7% (n = 1) reported a random outcome evaluation [22]. No study reported detailed information on baseline characteristics of animals, as well as details on hidden and blind allocation methods (risk bias = 100%). All studies had no incomplete results and selective results. None of the studies had a published pre-specified protocol. The effects of curcumin on hepatic fibrosis rats or mice models mainly include three aspects: The indicators related to liver cell structure and function mainly include ALT, AST, ALP, ALB, A/G, TBIL, bax protein, bcl-2 protein and index of liver. The main indicators for evaluating the degree of hepatic fibrosis include HA, LN, Collagen I, Collagen III, PCIII, PIIINP, IV-C, IL-6, and TNF-α, α-SMA, HYP, PDGF-BB, CTGF and TGF-β1. The indicators related to oxidative stress mainly include SOD, MDA and GSH-Px.

**Table 2:** SYRCLE’s RoB tool for each experimental animal studies.

Serial number	Study ID	Random sequence generation	Baseline characteristics	Random housing	Blinding (study team)	Random outcome assessment	Blinding (outcome assessors)	Incomplete outcome data	Selective outcome reporting
		Selection Bias		Performance Bias		Detection Bias		Attrition Bias	Reporting Bias
1	CWS2013 [10]	?	-	?	-	N	-	?	?
2	CWL2013 [11]	+	-	?	-	N	-	?	?
3	DSE2011 [12]	?	-	+	-	N	-	?	?
4	DYH2012 [13]	?	-	?	-	N	-	?	?
5	FV2010 [14]	?	-	+	-	N	-	?	?
6	GCS2009 [15]	?	-	?	-	N	-	?	?
7	HHY2009 [16]	?	-	?	-	N	-	?	?
8	HH2009 [17]	?	-	?	-	N	-	?	?
9	HHY2008 [18]	?	-	?	-	N	-	?	?
10	HJX2009 [19]	?	-	+	-	N	-	?	?
11	HLM2019 [20]	?	-	+	-	N	-	?	?
12	HN2007 [21]	+	-	+	-	N	-	?	?
13	HSM2006 [22]	+	-	+	-	+	-	?	?
14	HTH2009 [23]	+	-	?	-	N	-	?	?
15	HTH2008 [24]	+	-	+	-	N	-	?	?
16	HTHh2008 [25]	?	-	?	-	N	-	?	?
17	HXH2015 [26]	?	-	?	-	N	-	?	?
18	HXHh2015 [27]	?	-	?	-	N	-	?	?
19	JH2010 [28]	?	-	?	-	N	-	?	?
20	JWW2009 [29]	+	-	+	-	N	-	?	?
21	LHY2007 [30]	?	-	?	-	N	-	?	?
22	LJL2010 [31]	?	-	?	-	N	-	?	?
23	LYGg2002 [32]	?	-	?	-	N	-	?	?
24	LYG2002 [33]	?	-	?	-	N	-	?	?
25	LYG2005 [34]	?	-	?	-	N	-	?	?
26	LZX2019 [35]	?	-	?	-	N	-	?	?
27	MAMAMA2012 [36]	?	-	+	-	N	-	?	?
28	MEW2012 [37]	?	-	+	-	N	-	?	?
29	MJ2011 [38]	?	-	+	-	N	-	?	?
30	MMA2016 [39]	?	-	+	-	N	-	?	?
31	OYP2016 [40]	?	-	+	-	N	-	?	?
32	PCW2014 [41]	+	-	?	-	N	-	?	?
33	RLJ2019 [42]	?	-	+	-	N	-	?	?
34	RXF2011 [43]	?	-	?	-	N	-	?	?
35	SCJ2020 [44]	+	-	+	-	N	-	?	?
36	SESE2020 [45]	?	-	+	-	N	-	?	?
37	SJ2006 [46]	?	-	+	-	N	-	?	?
38	SJCc2008 [47]	?	-	?	-	N	-	?	?
39	SJC2008 [48]	?	-	+	-	N	-	?	?
40	SJC2007 [49]	?	-	+	-	N	-	?	?
41	SN2012 [50]	?	-	?	-	N	-	?	?
42	SSC2007 [51]	?	-	+	-	N	-	?	?
43	TFL2012 [52]	?	-	+	-	N	-	?	?
44	WJ2015 [53]	?	-	+	-	N	-	?	?
45	WLF2011 [54]	?	-	?	-	N	-	?	?
46	WRR2019 [55]	?	-	+	-	N	-	?	?
47	WTH2021 [56]	?	-	+	-	N	-	?	?
48	WXJ2015 [57]	?	-	?	-	N	-	?	?
49	WZK2020 [58]	?	-	+	-	N	-	?	?
50	XZY2018 [59]	?	-	?	-	N	-	?	?
51	YGR2007 [60]	?	-	+	-	N	-	?	?
52	YWFf2003 [61]	?	-	?	-	N	-	?	?
53	ZZD2009 [62]	?	-	?	-	N	-	?	?
54	ZY2015 [63]	?	-	+	-	N	-	?	?
55	ZXR2007 [64]	?	-	+	-	N	-	?	?
56	ZXH2010 [65]	?	-	+	-	N	-	?	?
57	ZCB2020 [66]	?	-	?	-	N	-	?	?
58	YZ2014 [67]	?	-	+	-	N	-	?	?
59	YYR2022 [68]	?	-	+	-	N	-	?	?

**Note:** +: low risk of bias; -: high risk of bias;?: unclear risk of bias; N: not applicable.

### The effect of curcumin on related indicators of liver cell structure and function in hepatic fibrosis rats or mice models

**Fig 2:**
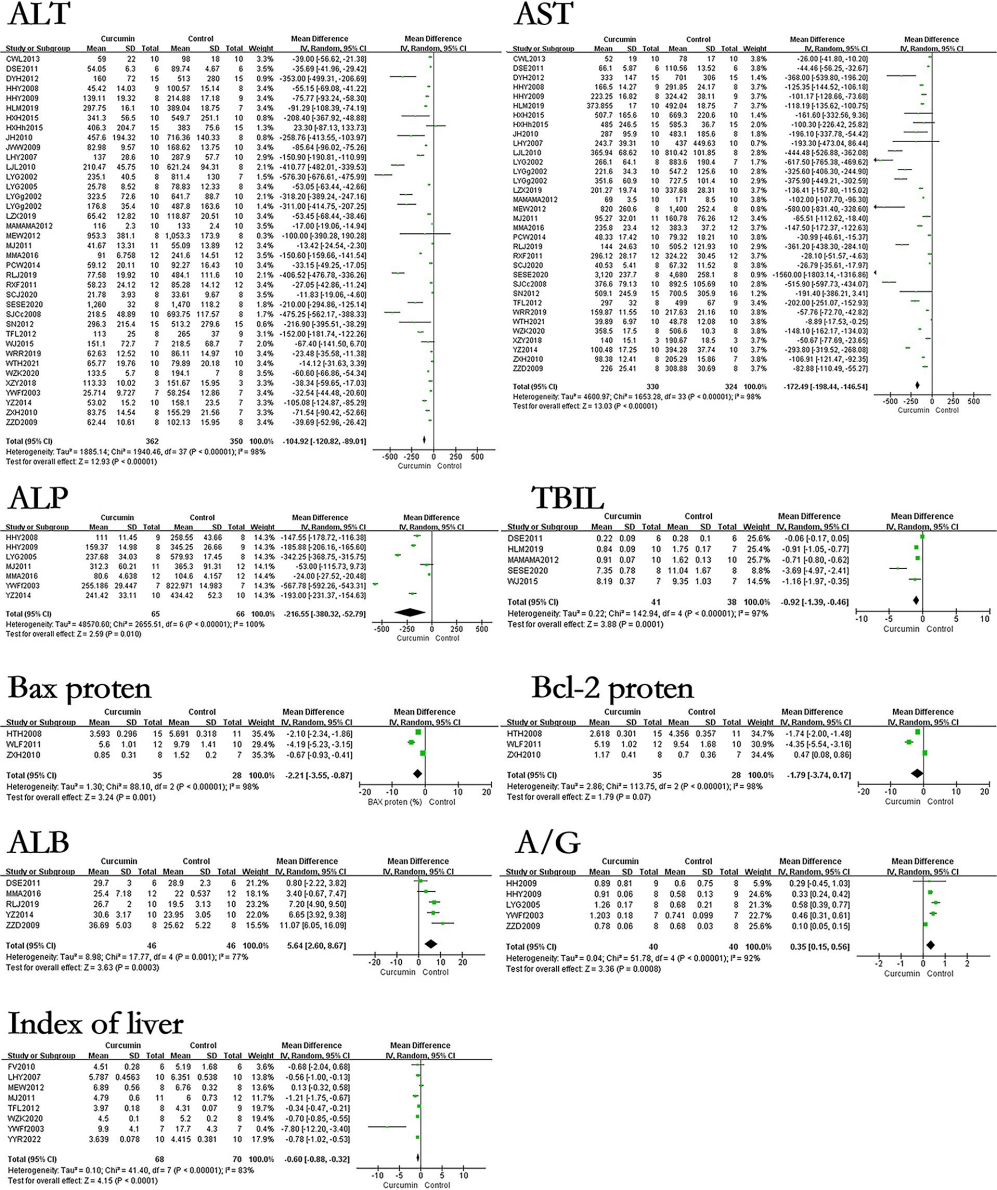
Forest plot of liver cell structure and function related indicators ALT, AST, ALP, TBIL, bax protein, bcl-2 protein, ALB, A/G, and Index of liver.

The results showed that curcumin can reduce the levels of ALT, AST, ALP, TBIL, bax protein and index of liver in hepatic fibrosis models, and improve the levels of ALB and A/G of the hepatic fibrosis models. However, the effect of curcumin on bcl-2 protein in the hepatic fibrosis models was not statistically significant. The results found that the use of curcumin can reduce the levels of ALT by 104.92U/L (p<0.00001), AST by 172.49U/L (p<0.00001), ALP by 216.55U/L (p<0.00001), TBIL by 0.92mg/dL (p<0.00001), bax protein by 2.21% (p = 0.001), index of liver by 0.60% (p<0.00001). The results also found that the use of curcumin can increase the levels of ALB by 5.64g/L (p = 0.0003), A/G by 0.35 (p = 0.0008). The forest map is presented in Fig 2.

### The effect of curcumin on relevant indicators related to evaluating the degree of hepatic fibrosis of hepatic fibrosis rats or mice models

**Fig 3:**
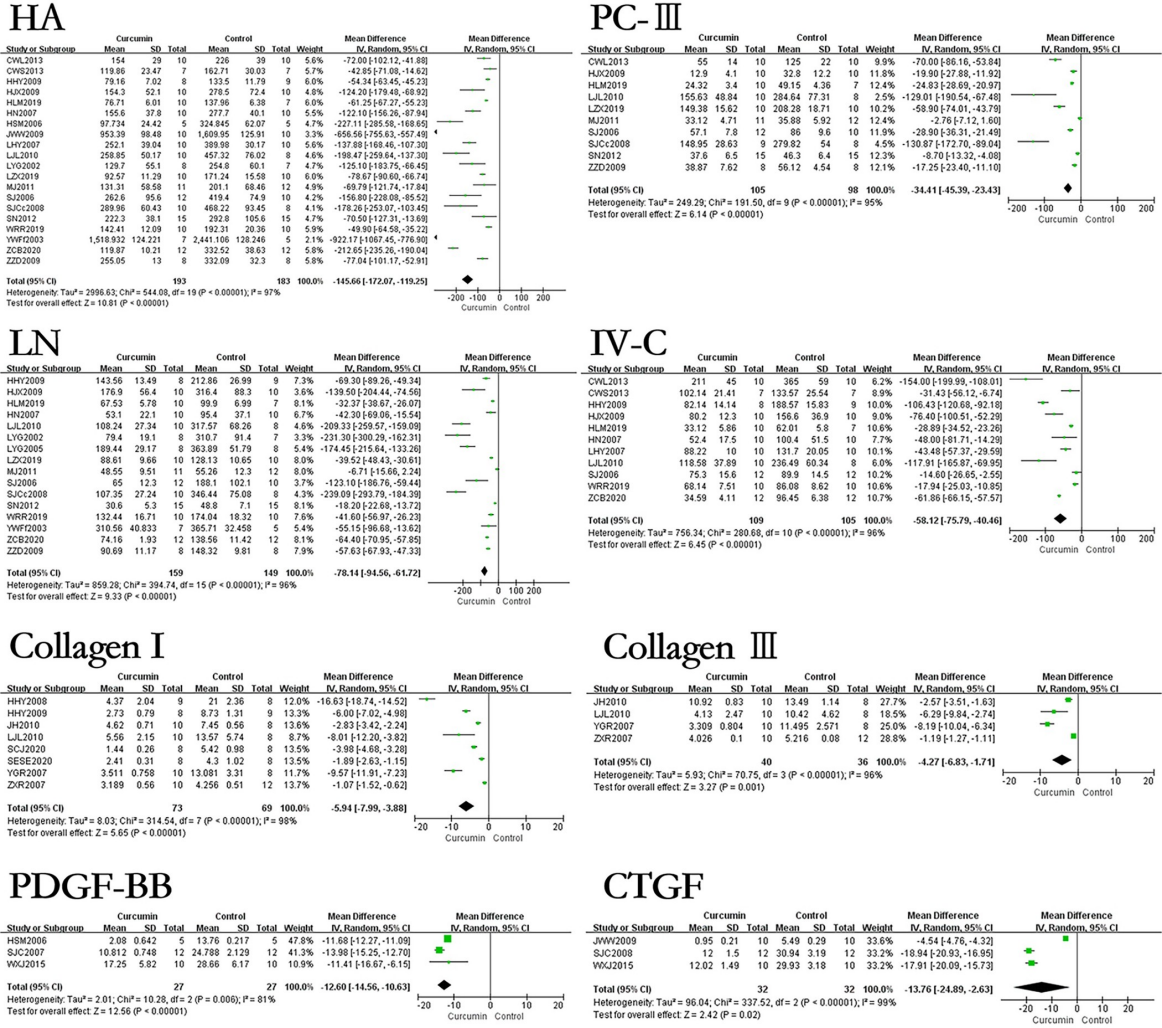
Forest plot of liver fibrosis degree related indicators HA, LN, PCIII, IV-C, Collagen I, Collagen III, PDGF-BB, and CTGF.

**Fig 4:**
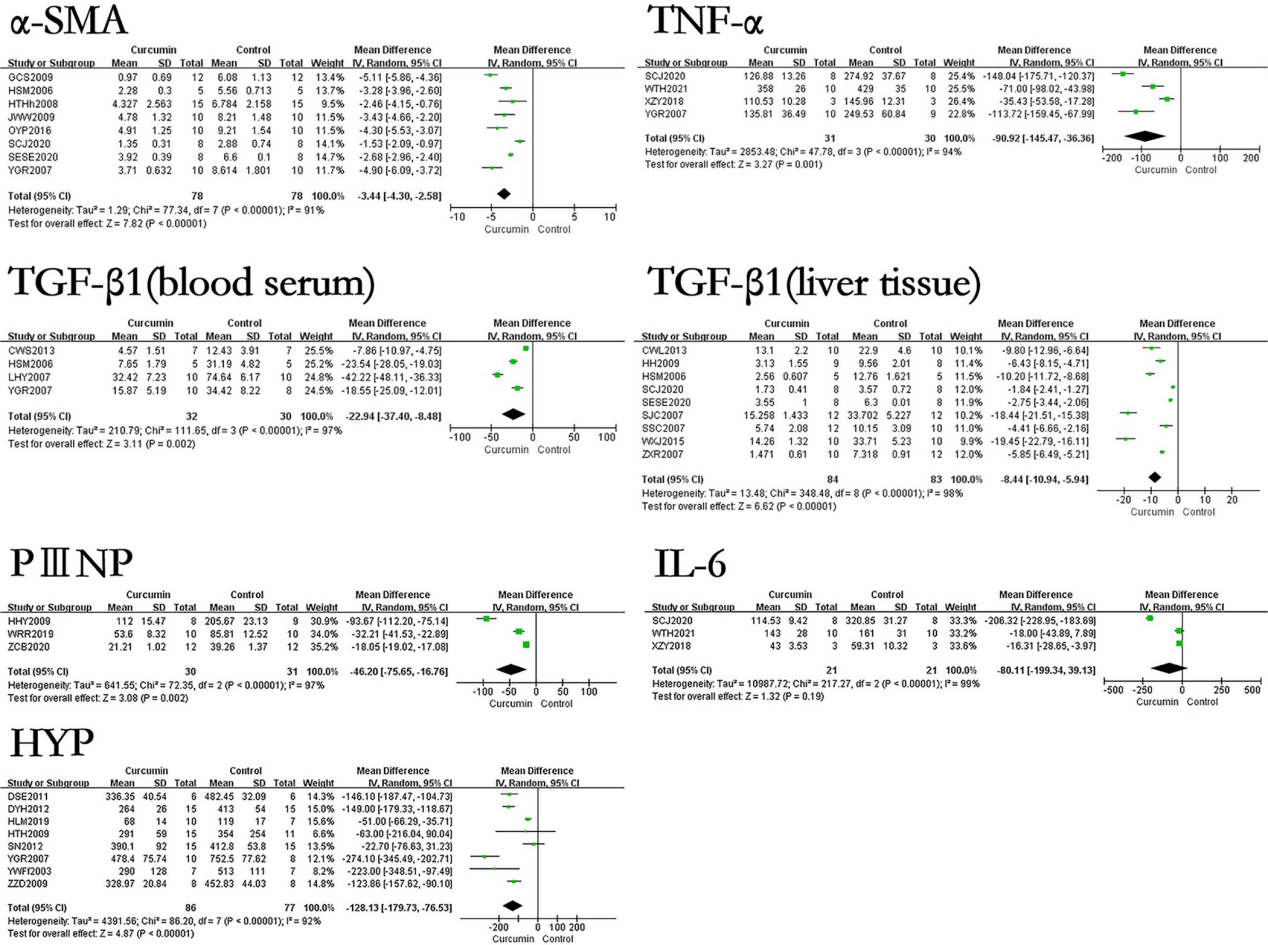
Forest plot of liver fibrosis degree related indicators α-SMA, TNF-α, TGF-β1(blood serum), TGF-β1(liver tissue), PIIINP, IL-6, and HYP.

The results showed that the use of curcumin can reduce the levels of HA by 145.66ng/ml (p<0.00001), LN by 78.14ng/ml (p<0.00001), PCIII by 34.41ng/ml (p<0.00001), IV-C by 58.12ng/ml (p<0.00001), PⅢNP by 46.20μg/L (p<0.00001), HYP by 128.13μg/g·liver (p<0.00001), TNF-α by 90.92ng/L (p = 0.001), TGF-β1 (blood serum) by 22.94ng/ml (p<0.00001). In terms of immunohistochemical results, the use of curcumin can decreased the levels of TGF-β1(liver tissue) by 8.44% (p<0.00001), Collagen I by 5.94% (p<0.00001), Collagen III by 4.27% (p = 0.001), PDGF-BB by 12.6% (p<0.00001), CTGF by 13.76% (p = 0.02), α-SMA by 3.44% (p<0.00001). However, the effect of curcumin on IL-6 in the hepatic fibrosis models was not statistically significant. The forest map is presented in Figs 3 and 4.

### The effect of curcumin on oxidative stress-related indicators in hepatic fibrosis rats or mice models

**Fig 5:**
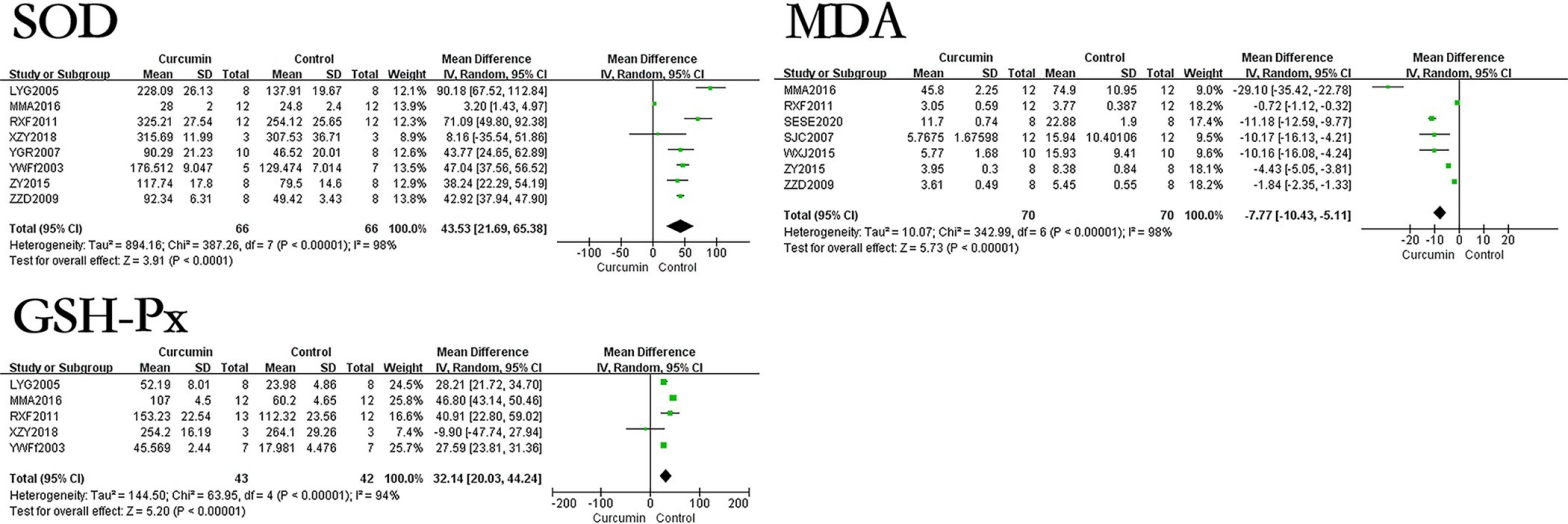
Forest plot of oxidative stress related indicators SOD, MDA, and GSH-Px.

The oxidative stress-related indicators in this study mainly include SOD, MDA, and GSH-Px.The results showed that curcumin can increase the levels of SOD and GSH-Px in hepatic fibrosis rats or mice models, and reduce MDA levels, it can increase SOD by 43.53U/mg (p<0.00001), GSH-Px by 32.14U/mg (p<0.00001), and reduce MDA by 7.77nmol/mg (p<0.00001). The forest map is presented in Fig 5.

### Sub study of rats and mice

**Fig 6:**
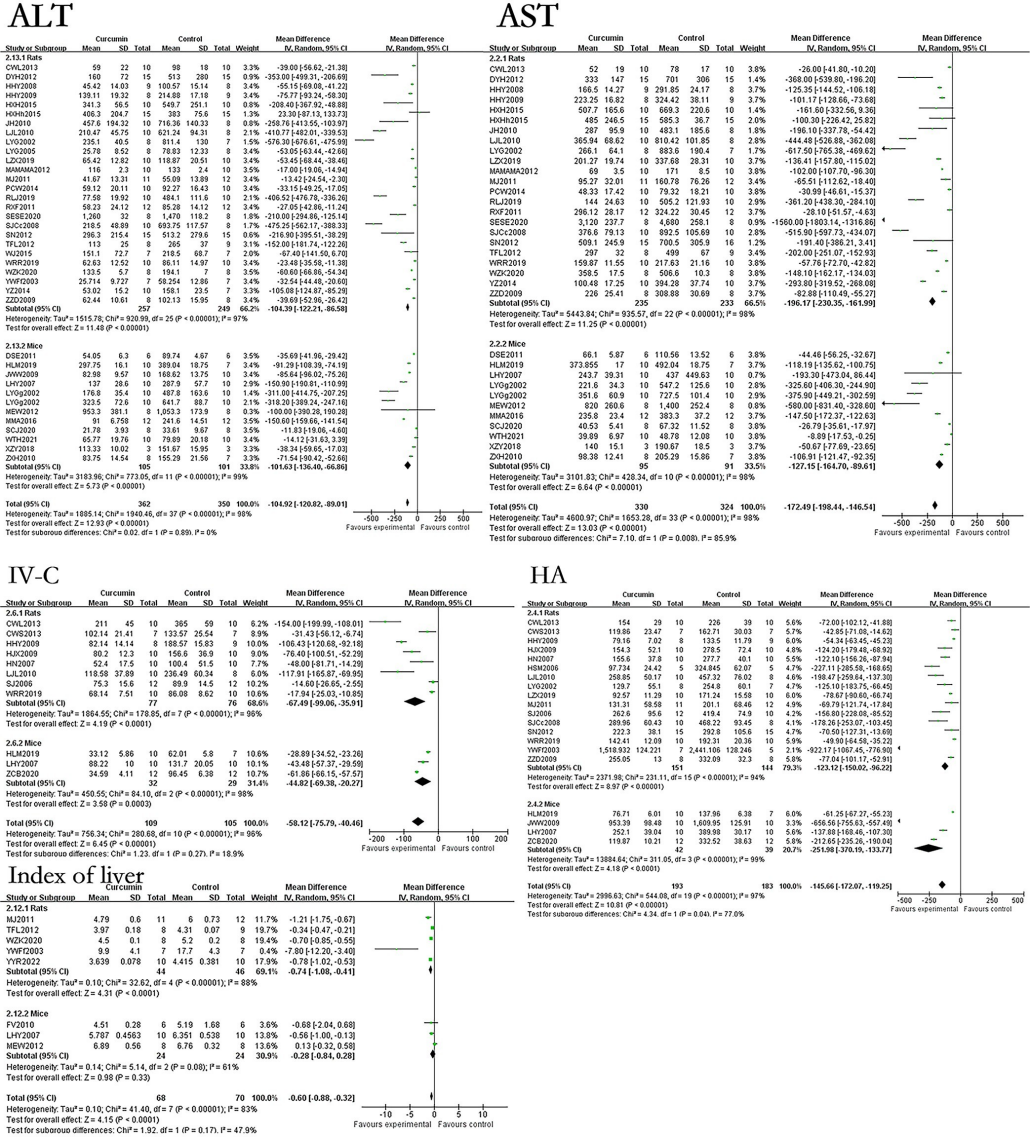
Forest plot of sub studies of rats and mice of AST, ALT, HA, IV-C, and Index of liver.

In order to study the effects of curcumin on different animal models of hepatic fibrosis, we classified the included literatures according to rats and mice. When there are less than 3 relevant literatures on outcome indicators for rats or mice, the outcome indicator will be excluded. In our included research literatures, the hepatic fibrosis models for A/G, Collagen III, and PDGF-BB immunohistochemistry results were all rats, while the hepatic fibrosis models for IL-6 were all mice. The research literatsure on AST, ALT, HA, IV-C, and index of liver includes three or more studies related to rats or mice. The forest map is presented in Fig 6.

According to the results, curcumin can reduce ALT by 104.39U/L (p<0.00001) in the hepatic fibrosis rats model and 101.63U/L (p<0.00001) in the hepatic fibrosis mice model. Curcumin can reduce AST by 196.17U/L (p<0.00001) in the hepatic fibrosis rats model and 127.15U/L (p<0.00001) in the hepatic fibrosis mice model. Curcumin can reduce HA by 123.12ng/ml (p<0.00001) in the hepatic fibrosis rats model and 251.98ng/ml (p<0.00001) in the hepatic fibrosis mice model. Curcumin can reduce IV-C by 67.49ng/ml (p<0.0001) in the hepatic fibrosis rats model and 44.82ng/ml (p = 0.0003) in the hepatic fibrosis mice model. Curcumin can reduce the index of liver of hepatic fibrosis rats model by 0.74% (p<0.0001), but its effect on the index of liver of hepatic fibrosis mice model is not statistically significant.

**Fig 7:**
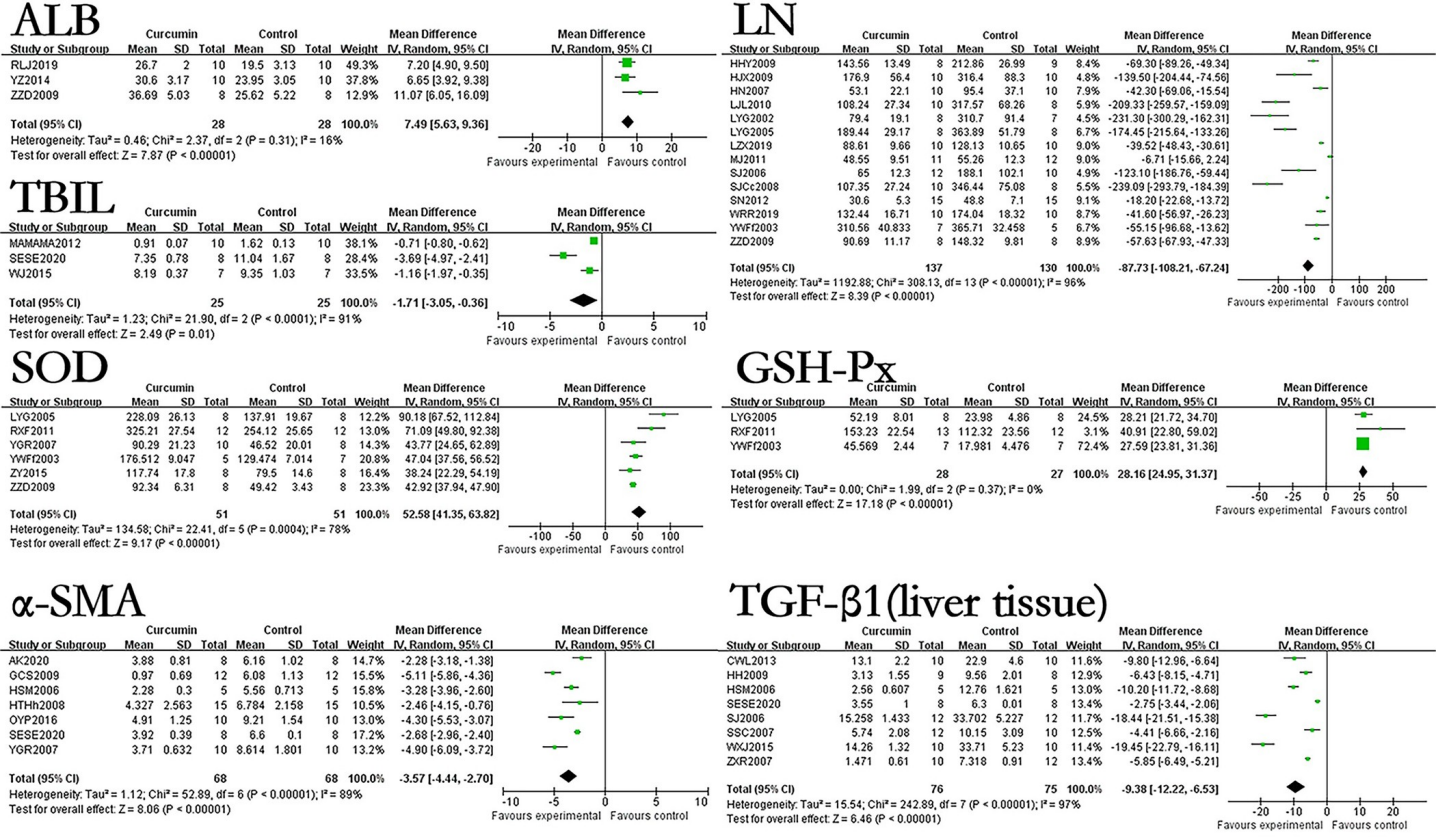
Forest plot of ALB, TBIL, LN, SOD, GSH-Px, α-SMA and TGF-β1 (liver tissue) of rats.

**Fig 8:**
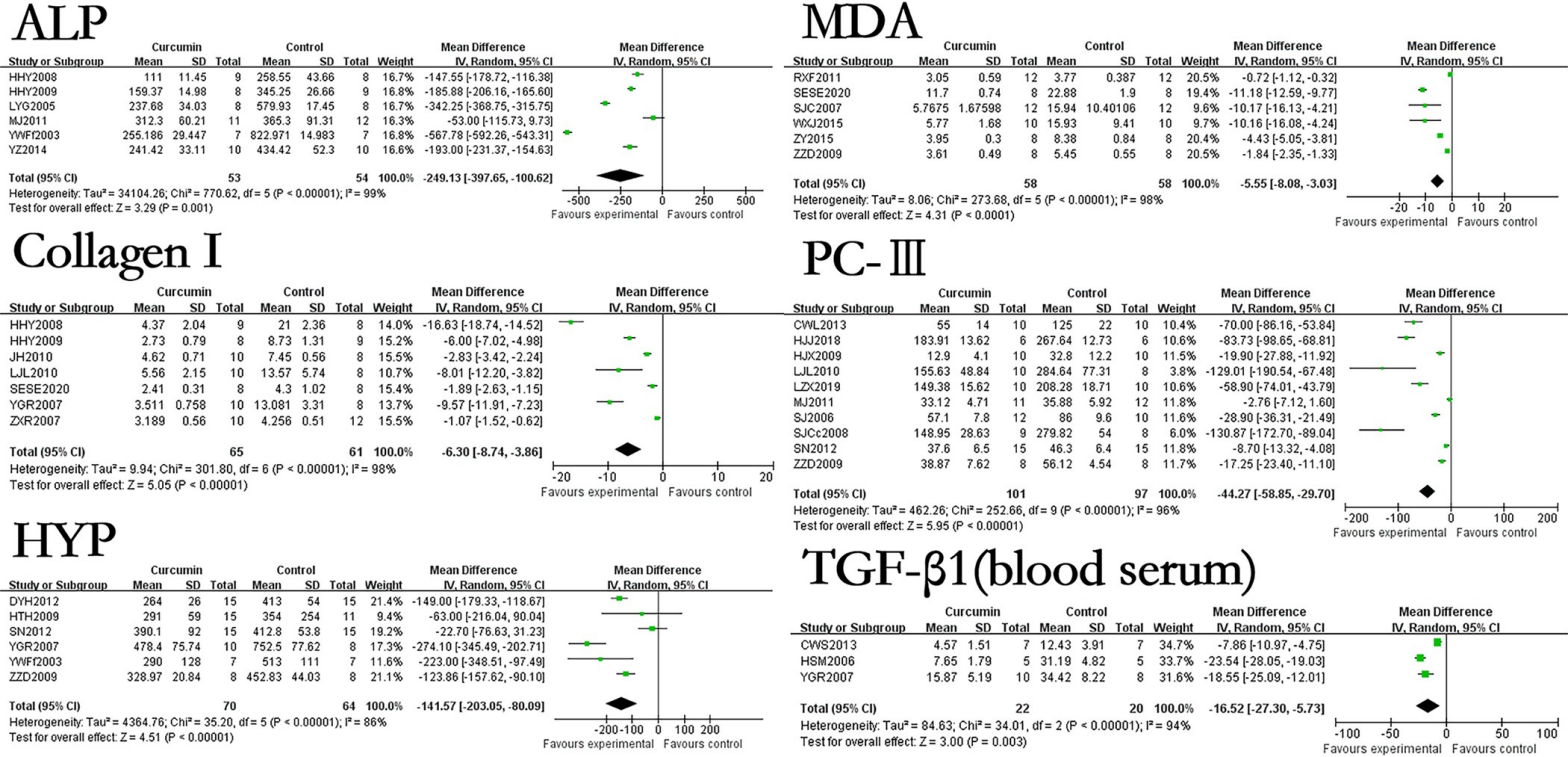
Forest plot of ALP, Collagen I, MDA, PCⅢ, HYP and TGF-β1 (blood serum) of rats.

In ALB, TBIL, LN, SOD, GSH-Px, α-SMA and HYP among the included literatures, only 2 articles were about hepatic fibrosis mice models. In ALP, Collagen I, MDA, PCⅢ, TGF-β1 (liver tissue) and TGF-β1 (blood serum) among the included literatures, only 1 article is about the hepatic fibrosis mice model. We removed the mice related studies and observed the impact on the research results again, the forest map is presented in Figs 7 and 8.

The results showed that after removing mice related studies, the use of curcumin could increase ALB by 7.49g/L (p<0.00001), reduce TBIL by 1.71mg/dL (p = 0.01), decrease LN by 87.73ng/ml (p<0.00001), increase SOD by 52.58U/mg (p<0.00001) and increase GSH-Px by 28.16U/mg (p<0.00001), reduce the immunohistochemical results of α-SMA by 3.57% (p<0.00001), reduce the immunohistochemical results of TGF-β1(liver tissue) by 9.38% (p<0.00001), reduce ALP by 249.13U/L (p = 0.001), reduce the immunohistochemical results of Collagen I by 6.3% (p<0.00001), reduce MDA by 5.55nmol/mg (p<0.0001), reduce PCIII by 44.27μg/L (p<0.00001), reduce HYP by 141.57μg/g·liver (p<0.00001), reduce TGF-β1(Blood serum) by 16.52ng/ml (p<0.00001). These results showed no significant difference compared to previous studies on removing mice.

**Fig 9:**
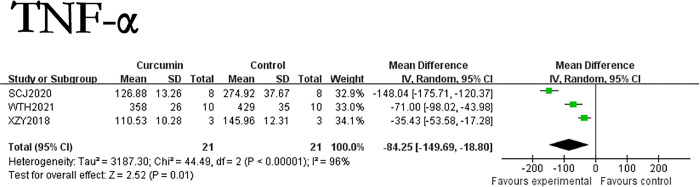
Forest plot of TNF-α of mice.

Among the included research literatsure on TNF-α, only one was a hepatic fibrosis rats model. We removed the rats related studies and observe the research results again. It was found that the use of curcumin can reduce the TNF-α of hepatic fibrosis mice model by 84.25ng/L (p = 0.001). The forest map is presented in Fig 9, there is no significant difference in the results compared to before removing the relevant studies.

### Sensitivity analysis and publication bias of curcumin on relevant indicators in hepatic fibrosis rats or mice models

**Fig 10:**
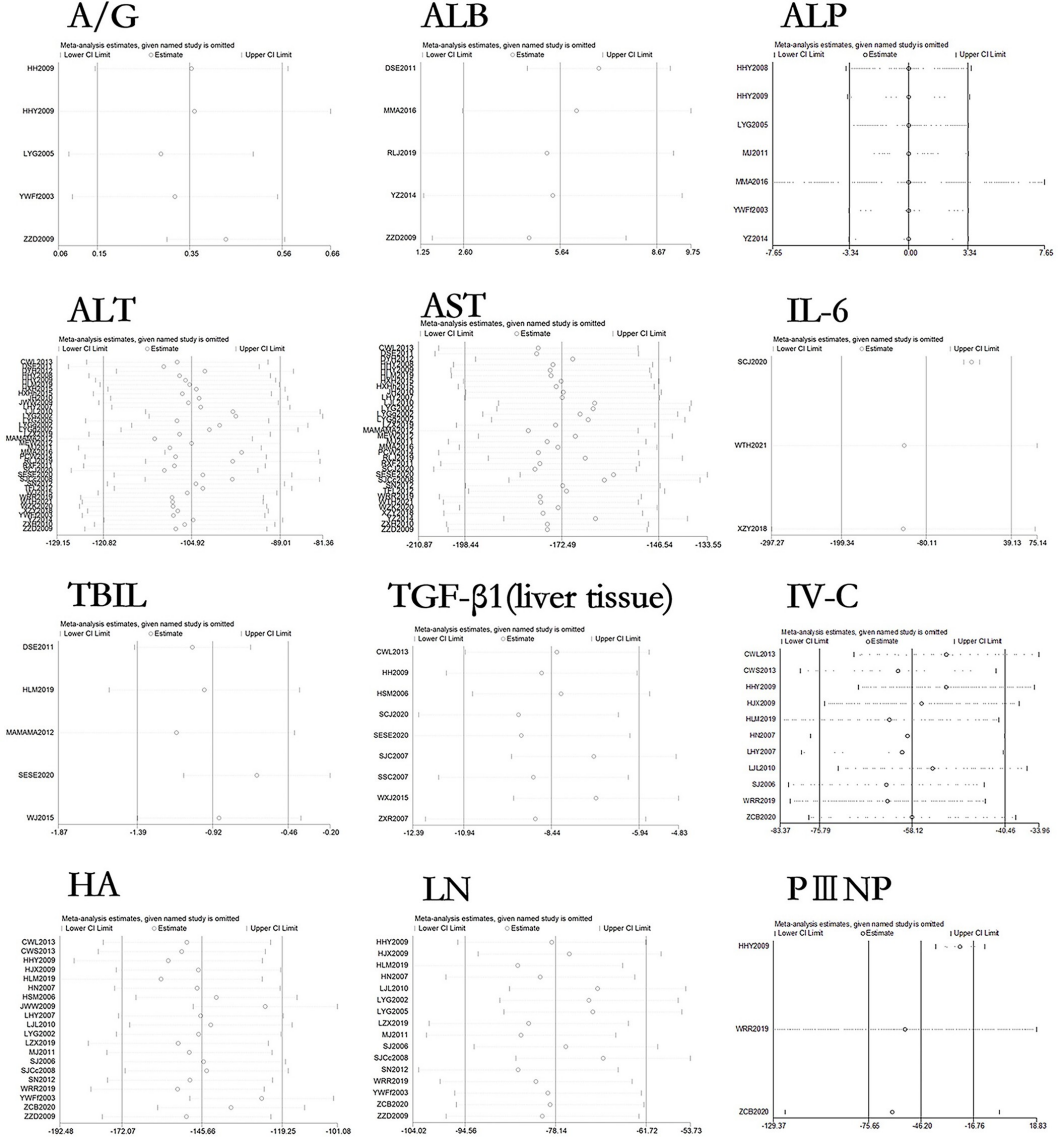
Sensitivity analysis of AG, ALB, ALP, ALT, AST, IL-6, TBIL, TGF-β1 (liver tissue), IV-C, HA, LN, and PIIINP in liver fibrosis rat or mouse models.

**Fig 11:**
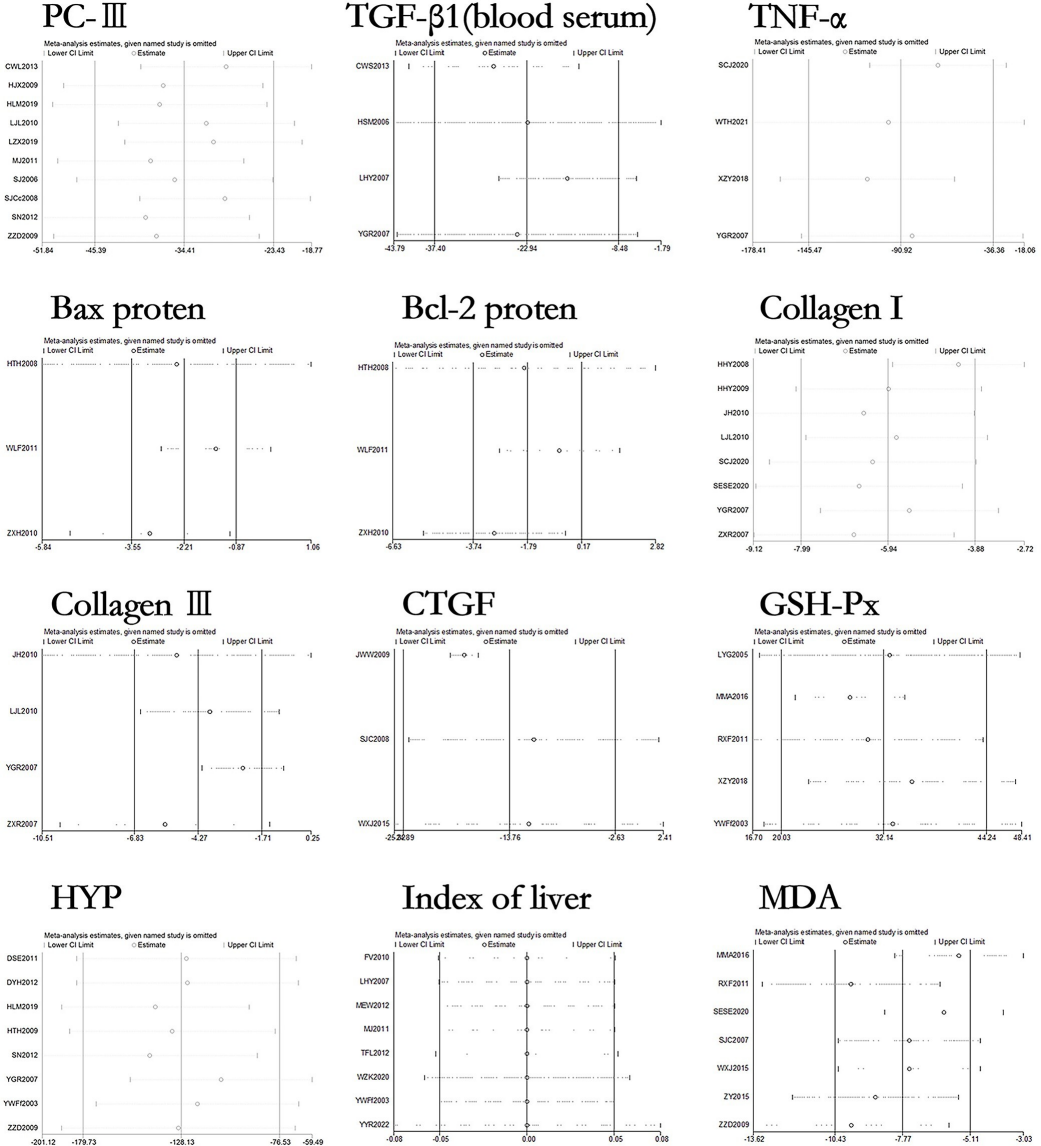
Sensitivity analysis of PCIII, TGF-β1 (blood serum), TNF-α, bax protein, bcl-2 protein, Collagen I, Collagen III, CTGF, GSH-Px, HYP, index of liver and HYP in liver fibrosis rat or mouse models.

**Fig 12:**
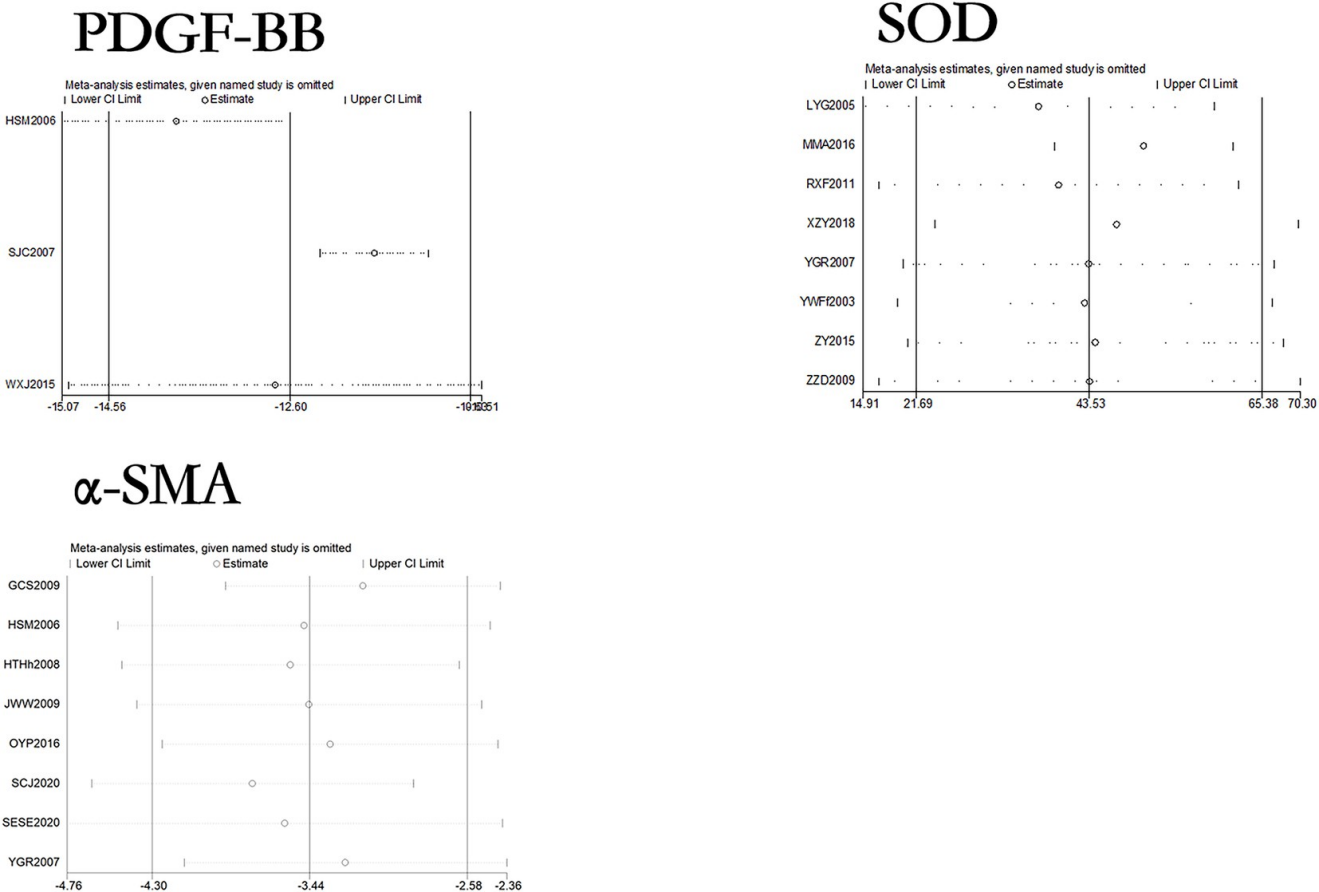
Sensitivity analysis of PDGF-BB, SOD, and α-SMA in liver fibrosis rat or mouse models.

The sensitivity analysis results of 27 outcome indicators in the liver fibrosis models are shown in Figs 10–12. After systematically excluding each study, the author found that the comprehensive effect of curcumin on liver fibrosis in rodent models did not reverse. The results indicate that the data analyzed in this meta-analysis is relatively stable and reliable.

We used the Begg rank correlation method and Egger linear regression method in Stata 12.0 to further evaluate publication bias. In the Begg test, the P-values of all research factors are greater than 0.05, and no publication bias was detected. In Egger tests with relatively high sensitivity, ALT (Egger’s test t = -4.89, Q = 1940.458, p<0.05), AST (Egger’s test t = -3.25, Q = 1653.281, p<0.05), HA (Egger’s test t = -3.76, Q = 544.077, p<0.05), LN (Egger’s test t = -3.44, Q = 394.739, p<0.05), PCIII (Egger’s test t = -3.07, Q = 191.502, p<0.05), SOD (Egger’s test t = 2.72, Q = 387.263, p<0.05), Collagen I (Egger’s test t = -3.33, Q = 314.545, p<0.05) and TGF-β1 (liver tissue) (Egger’s test t = -3.04, Q = 348.479, p<0.05), the results indicate that there is publication bias in all the outcome indicators mentioned above.

We further used the trim and fill method to observe whether the outcome indicators of the aforementioned biomarkers would be reversed. No virtual data was added after using the trim and fill method to analyze the outcome indicators of ALT, AST, LN, PC-III, SOD, and Collagen I, the random effect results were ALT(SMD = -104.915, 95%CI[-120.821, -89.010]), AST(SMD = -172.491, 95%CI[-198.444, -146.538]), LN(SMD = -78.139, 95%CI[-94.559, -61.720]), PCIII(SMD = -34.411, 95%CI[-45.395, -23.427]), SOD(SMD = 43.531, 95%CI[21.685, 65.377]) and Collagen I(SMD = -5.936, 95%CI[-7.995, -3.878]). This indicates that although there is publication bias in the above indicators, the outcomes are still robust.

We conducted the trim and fill method analysis on the outcome measures of HA and TGF-β1 (liver tissue). The Stata 12.0 used the Linear method to estimate the number of missing studies for HA after 5 iteratsions as 6. After adding data from 6 virtual studies and reanalyzing all studies, the final results showed heterogeneity testing: Q = 9067.451, p<0.05. The Stata 12.0 used the Linear method to estimate the number of missing studies for TGF-β1 (liver tissue) after 2 iteratsions as 1. After adding data from 1 virtual studies and reanalyzing all studies, the final results showed heterogeneity testing: Q = 1733.559, p<0.05. Both indicators showed no reversal compared to the random effect results before addition, indicating that although there was publication bias, the outcome remained robust.

## Discussion

The results showed that curcumin can reduce the levels of ALT, AST, ALP, TBIL, bax protein, and index of liver in hepatic fibrosis models.It can also reduce HA, LN, Collagen I, Collagen III, PCIII, PIIINP, IV-C, TNF-α, α-SMA, HYP, PDGF-BB, CTGF, TGF-β1 and MDA, and increase the levels of ALB, A/G, SOD, and GSH-Px in the hepatic fibrosis models. However, the effects of curcumin on bcl-2 protein, IL-6, and mice index of liver in the hepatic fibrosis models were not statistically significant.

Hepatic fibrosis is a pathological repair process in which multiple pathogenic factors stimulate and activate HSC, leading to abnormal proliferatsion of connective tissue in the liver, destruction of liver structure, and abnormal physiological function changes. When the structure and function of liver cells are damaged, it can cause liver cell swelling, increased index of liver, and increased membrane permeability, leading to the infiltratsion of permeable enzymes into the blood [71]. ALT and AST are the most sensitive indicators reflecting the stability of liver cell membranes. Index of liver is an important indicator of inflammatory lesions in liver cells. Apoptosis is controlled by two types of genes: pro apoptotic and anti apoptotic. Bax is a pro apoptotic factor, while bcl-2 is an anti apoptotic factor. The downregulation of Bax/Bcl-2 ratio is a key factor determining cell apoptosis [72]. In the hepatic fibrosis models, curcumin can significantly reduce the levels of ALT, AST, index of liver, and bax protein, and alleviate liver damage by maintaining liver cell membrane stability and reducing liver cell apoptosis. ALP and TBIL mainly reflect the excretion, secretion, and detoxification functions of the liver. When hepatic fibrosis occurs, widespread deposition of ECM in the liver leads to dysfunction of liver cells in extracting and excreting bilirubin. ALB is an important indicator reflecting the synthetic function of liver cells [73]. As liver parenchymal damage worsens, serum albumin gradually decreases while globulin gradually increases, and the A/G ratio gradually decreases. In the hepatic fibrosis models, curcumin can reduce ALP and TBIL content, increase ALB and A/G levels, and improve liver metabolism and synthesis function.

After being affected, damaged or necrotic by pathogenic factors, liver cells release a large amount of soluble cytokines and activate HSC [74]. Among numerous cytokines, PDGF and TGF-β1 are closely associated with liver fibrosis, with the former being the strongest known HSC proliferatsion promoting factor and the latter being the strongest fibroblast promoting factor [49]. In addition, in stimulating stromal cell proliferatsion and ECM synthesis, CTGF exhibits correlation with TGF-β1 similar biological functions [75]. HA is a protein polysaccharide synthesized by HSC. During the process of liver fibrosis, the gradual increase in the degree of damage to hepatic sinusoidal endothelial cells leads to a decrease in their ability to absorb and metabolize HA, resulting in the gradual deposition of HA in the serum [76]. LN is a large non collagenous structural glycoprotein synthesized by endothelial cells, participating in the support of the extracellular skeleton [77]. When cells are damaged or liver tissue undergoes fibrosis, it is released into the serum. In liver injury, TNF-α can promote both inflammatory response and proliferatsion and differentiation of stromal cells, as well as ECM synthesis [78]. α-SMA is a specific marker protein for HSC activation and transformation into myofibroblasts [79]. When liver fibrosis occurs, there will be a large amount of abnormal deposition of ECM, which is mainly composed of collagen. In the early stage of liver fibrosis, it mainly involves an increase in type IV collagen, and when liver fibrosis progresses to cirrhosis, it is mainly an increase in type I and III collagen, especially type I [80]. PCIII is the main component of collagen in liver ECM, PIIINP is a detection indicator that directly reflects ECM reconstruction during hepatic fibrosis [81], and HYP is a non essential amino acid that constitutes collagen in the body. The combined detection of these biomarkers is beneficial for the early diagnosis of hepatic fibrosis, and can indirectly reflect the synthesis status of collagen in the tissue and the degree of hepatic fibrosis, which is positively correlated with the level and degree of hepatic fibrosis activity. In the hepatic fibrosis models, curcumin can significantly reduce HA, LN, Collagen I, Collagen III, PCIII, PIIINP, IV-C, TNF-α, α-SMA, HYP, PDGF-BB, CTGF and TGF-β1 to alleviate inflammatory response, inhibit HSC activation and proliferatsion, and regulate the imbalance of ECM synthesis and degradation.

Oxidative stress refers to the pathological state of imbalance between reactive oxygen species or nitrogen produced by aerobic metabolism and antioxidant defense and elimination [82]. MDA is a product of lipid peroxidation, and its content not only reflects the degree of damage caused by lipid peroxidation, but also reflects the degree of damage caused by free radicals to liver cells. SOD has the function of clearing oxygen free radicals. When liver disease occurs, its detoxification function decreases. Ischemia and hypoxia can cause a decrease in SOD synthesis, leading to an increase in oxygen free radicals [83]. Its inhibitory effect on hepatic fibrosis is closely related to reducing tissue oxidative stress damage. GSH-Px is also an important antioxidant for clearing free radicals in the body, so its level changes are usually used as an important indicator for judging the production of oxygen free radicals and tissue damage [84]. In the hepatic fibrosis models, curcumin can reduce MDA levels and increase SOD and GSH-Px content, reducing tissue oxidative stress damage by clearing oxygen free radicals.

This study reported significant heterogeneity among different results. We systematically validated the sensitivity analysis of 27 outcome indicators in the liver fibrosis model using a row by row method, and the results showed no reversal, indicating the relative stability and credibility of the data. In the Begg test, the P-values of all study factors were greater than 0.05, and no publication bias was detected. In the relatively sensitive Egger assay, although there were publication biases in ALT, AST, HA, LN, PCIII, SOD, Collagen I, and TGF-β1 (liver tissue) indicators, we observed the above biomarkers using pruning and filling methods and found that the results were still robust. Considering moderate heterogeneity observed in the data synthesized using a random effects model, we conducted subgroup analysis to adjust for confounding factors and explore potential sources of heterogeneity. We conducted subgroup analysis on the included literature based on the different categories of rats and mice, and the subgroup analysis results showed no significant difference compared to the previous comprehensive analysis results. In some of the included research literature, the dosage of curcumin was divided into multiple levels, and we selected the study results with the highest dosage included in the study. The analysis results indicate that the dose of curcumin exhibits a dose-response relationship with increasing dose at different doses. However, due to significant differences in the distribution of doses included in the study, we are currently unable to classify the doses. In addition, there are insufficient reports on randomization procedures, animal baseline characteristics, and group concealment in the literature included in this study, which may be the reason for heterogeneity and bias in the meta-analysis results.

## Conclusion

The analysis results show that in terms of liver cell structure and function, curcumin can inhibit abnormal proliferation of liver connective tissue and structural damage by maintaining the stability of liver cell membrane, and reduce liver cell apoptosis to alleviate liver damage and improve liver metabolism and synthesis functions, such as excretion, secretion, detoxification, etc. In terms of the degree of hepatic fibrosis, curcumin can alleviate the degree of hepatic fibrosis by reducing inflammatory response, inhibiting HSC activation and proliferatsion, and regulating the imbalance of ECM synthesis and degradation. In terms of oxidative stress, curcumin can reduce tissue peroxidation damage by clearing oxygen free radicals. This provides meaningful guidance for clinical practice. In addition, during the research process, it was found that the comprehensive application of the above serological and biochemical indicators has guiding significance for determining the presence of liver fibrosis and distinguishing between liver fibrosis and cirrhosis. For example, if the four serum indicators of liver fibrosis (HA, PIIINP, IV-C, and LN) are higher than the normal reference value, it can be considered that liver fibrosis has occurred. However, serological and biochemical indicators have no direct guiding significance for the specific staging of intermediate degrees of liver fibrosis. Due to different etiologies, the development of chronic liver disease into hepatitis, liver fibrosis, cirrhosis, and liver cancer is a dynamic process that makes it difficult to make an accurate diagnosis based on a single examination result. Therefore, early and effective clinical intervention is crucial for the delay and reversal of liver fibrosis, and the combination of multiple indicators and dynamic measurements may be more helpful in determining the trend and treatment effect of liver fibrosis.

However, due to the significant physiological and pathological differences between rodents and humans, rodent models cannot fully summarize the complexity of human liver fibrosis, and the translation of preclinical results into clinical environments may be limited as a result. Therefore, the clinical relevance and applicability of the results of this study in the human population are still uncertain, and caution should be exercised when translating them into clinical settings based on the evaluation of animal experimental systems. This study collected relevant literatsure as comprehensively as possible, and strictly screened literatsure according to inclusion and exclusion criteria and literatsure quality to avoid retrieval bias to the greatest extent possible. However, some literatsure could only be discarded due to different effect indicators or inability to calculate original data. In addition, language bias, publication bias, and reporting bias may all have an impact on the results of this study. Due to limitations in research types, this study only searched publicly published literatsure and cannot exclude unpublished studies that may affect the results of this study. The quality score of the literatsure included in this study is relatively low, with significant heterogeneity. The accuracy of the conclusions still needs to be verified through higher quality and larger sample animal experiments.

## Supporting information

S1 File PRISMA 2020 checklist. (PDF)

S2 File PROSPERO registration document: Animal review. (PDF)
