# Supplementary material for: The intervention of curcumin on rodent models of hepatic fibrosis: A systematic review and meta-analysis
Source: PLoS One. 2024 May 23;19(5):e0304176. doi: 10.1371/journal.pone.0304176 (PMC11115266; doi:10.1371/journal.pone.0304176)
Supplement: S2 File — (PDF) [file pone.0304176.s002.pdf]

Animal review

1. * Review title. [1 change]

Give the working title of the review. This must be in English. The title should have the interventions or exposures being reviewed and the associated health or social problems.

The intervention of curcumin on rodent models of hepatic fibrosis : A systematic review and meta-analysis

2. Original language title. [1 change]

For reviews in languages other than English, this field should be used to enter the title in the language of the review. This will be displayed together with the English language title.

姜黄素对啮齿类动物肝纤维化模型的干预作用：系统综述和荟萃分析

3. * Anticipated or actual start date.

Give the date when the systematic review commenced, or is expected to commence.

10/11/2023

4. * Anticipated completion date.

Give the date by which the review is expected to be completed.

10/03/2024

5. * Stage of review at time of this submission.

Indicate the stage of progress of the review by ticking the relevant Started and Completed boxes. Additional information may be added in the free text box provided.

Please note: Reviews that have progressed beyond the point of completing data extraction at the time of initial registration are not eligible for inclusion in PROSPERO. Should evidence of incorrect status and/or completion date being supplied at the time of submission come to light, the content of the PROSPERO record will be removed leaving only the title and named contact details and a statement that inaccuracies in the stage of the review date had been identified.

This field should be updated when any amendments are made to a published record and on completion and publication of the review.

The review has not yet started: No

Review stage	Started	Completed
Preliminary searches	Yes	No
Piloting of the study selection process	Yes	No
Formal screening of search results against eligibility criteria	No	No
Data extraction	No	No

Review stage	Started	Completed
Risk of bias (quality) assessment	No	No
Data analysis	No	No

Provide any other relevant information about the stage of the review here.

6. * Named contact.

The named contact acts as the guarantor for the accuracy of the information presented in the register record.

Yan Leng

Email salutation (e.g. "Dr Smith" or "Joanne") for correspondence:
Professor Leng

7. * Named contact email.

Enter the electronic mail address of the named contact.

ccucm_ly@outlook.com

8. * Named contact address.

PLEASE NOTE this information will be published in the PROSPERO record so please do not enter private information

Enter the full postal address for the named contact.

Department of Hepatology, Changchun Changchun University of Chinese Medicine Hospital, Jilin province, China

9. Named contact phone number

Enter the telephone number for the named contact, including international dialling code.

18243942968

10. * Organisational affiliation of the review.

Full title of the organisational affiliations for this review and website address if available. This field may be completed as 'none' if the review is not affiliated to any organisation.

The Changchun University of Chinese Medicine Hospital's Hepatology Department

Organisation web address:
https://y.ccucm.edu.cn/

11. * Review team members and their organisational affiliations.

Give the personal details and the organisational affiliations of each member of the review team. Affiliation refers to groups or organisations to which review team members belong. **NOTE: email and country are now mandatory fields for each person.**

Professor Yan Leng. The Changchun University of Chinese Medicine Hospital's Hepatology Department
Mr Yun-Hang Chu. Changchun University of Chinese Medicine
Miss Bing-Yao Pang. The Changchun University of Chinese Medicine Hospital
Mr Ming Yang. The Changchun University of Chinese Medicine Hospital
Qi Meng. The Changchun University of Chinese Medicine Hospital

12. * Funding sources/sponsors.

Give details of the individuals, organisations, groups or other legal entities who take responsibility for initiating, managing, sponsoring and/or financing the review. Any unique identification numbers assigned to the review by the individuals or bodies listed should be included.

None

Grant number(s)
None

13. * Conflicts of interest.

List any conditions that could lead to actual or perceived undue influence on judgements concerning the main topic investigated in the review.

None

14. Collaborators.

Give the name, affiliation and role of any individuals or organisations who are working on the review but who are not listed as review team members.

15. * Review question. [1 change]

Give details of the question to be addressed by the review, clearly and precisely.

Does intervention with curcumin improve hepatic injury related physicochemical indicators in a rodent model of hepatic fibrosis?

Context and rationale
From the clinical and pathological evolution of many chronic hepatic diseases, especially chronic hepatitis, hepatic fibrosis is an inevitable stage for chronic hepatic disease to develop into cirrhosis. A 2020 epidemiological study showed that as of 2017, the annual mortality rate of chronic hepatic disease in the United States was 12.86 ‰, and cirrhosis was 7.96 ‰. The reversibility of hepatic fibrosis brings hope for treatment, but the pathogenesis of hepatic fibrosis is still not fully understood, and there is still no recognized standard for anti hepatic fibrosis. Curcumin is a natural active ingredient extracted from the roots and stems of plants such as turmeric. Many researchers have studied the function and molecular mechanism of curcumin in animal models. Animal research, as the basis of clinical research, is of great significance to human health.

16. * Searches.

Give details of the sources to be searched, and any restrictions (e.g. language or publication period). The full search strategy is not required, but may be supplied as a link or attachment.

We expect to use PubMed, EMBASE, Cochrane Central Registry of Controlled Trials, Web of Science, CNKI, CBM, wanfang, VIP, and ClinicalTrials.gov, the time range is from the date the database is created to November 10 of the 2023. The search keywords included the following three sections: 1) curcumin; 2) hepatic fibrosis/liver fibrosis; 3) rat or mouse, with and links between keywords.

17. URL to search strategy. [1 change]

Give a link to the search strategy or an example of a search strategy for a specific database if available (including the keywords that will be used in the search strategies).

Do not make this file publicly available until the review is complete

18. * Human disease modelled. [1 change]

Give a short description of the disease, condition or healthcare domain being modelled.

Hepatic fibrosis.

19. * Animals/population. [1 change]

Give summary criteria for the animals being studied by the review, e.g. species, sex, details of disease model. Please include details of both inclusion and exclusion criteria.

Inclusion criteria:
Rat or mice models of hepatic fibrosis; the method of preparation of the model must be recognized.

Exclusion criteria:
1)The study involved experimental animals other than rats and mice.
2)Ex vivo, in vitro and in silico models.

20. * Intervention(s), exposure(s). [1 change]

Give full and clear descriptions of the nature of the interventions or the exposures to be reviewed (e.g. dosage, timing, frequency). Please include details of both inclusion and exclusion criteria.

Inclusion criteria:
The drug used must be curcumin or a curcumin preparation. The experimental group was not given additional drugs that might affect the liver.

Exclusion criteria:
The experimental group or the control group involved the use of other drugs.

21. * Comparator(s)/control. [2 changes]

Where relevant, give details of the type(s) of control interventions against which the experimental condition(s) will be compared (e.g. another intervention or a non-exposed control group). Please include details of both inclusion and exclusion criteria.

Inclusion criteria:
The control group was given no drugs or only a placebo.

Exclusion criteria:
The control group involved the use of other drugs.

22. * Study designs to be included. [2 changes]

Give details of the study designs eligible for inclusion in the review. If there are no restrictions on the types of study design eligible for inclusion, or certain study types are excluded, this should be stated. Please include details of both inclusion and exclusion criteria.

Inclusion criteria:
We will use a controlled study with separate treatment groups to evaluate the effect of curcumin intervention on liver fibrosis in rodents.

Exclusion criteria:
A controlled study of the non separate treatment groups.

23. Other selection criteria or limitations applied. [1 change]

Give details of any other inclusion and exclusion criteria, e.g. publication types (reviews, conference abstracts), publication date, or language restrictions.

Exclusion criteria:
The article is a meta-analysis, systematic review, or incomplete communication information.

24. * Outcome measure(s). [1 change]

Give detail of the outcome measures to be considered for inclusion in the review. Please include details of both inclusion and exclusion criteria.

Inclusion criteria:

Inclusion criteria: Relevant physicochemical outcome indicators of curcumin intervention on hepatic injury in rodents with hepatic fibrosis, including hepatic fibrosis indicators (such as HA, LN, PC-III, and IV-C), hepatic cell structure and function indicators (such as ALT, AST, ALP, ALB, A/G, TP, and TBIL), and oxidative stress indicators (such as SOD, MDA, and GSH Px).

Exclusion criteria:

Report on results unrelated to the effect of hepatic injury in rodents with hepatic fibrosis.

25. N/A.

This question does not apply to systematic reviews of animal studies for human health submissions.

26. * Study selection and data extraction. [2 changes]

Procedure for study selection

We will filter the title, abstract, and full text, with two individuals making independent study selection. When any differences arise, they can be resolved through discussion or third-party intervention until a consensus is reached.

Prioritise the exclusion criteria

1)The article is a meta-analysis, systematic review, or incomplete communication information.

2) The study involves experimental animals other than rats and mice.

3) Ex vivo, in vitro and in silicon models.

4)The experimental group or control group involves the use of other drugs.

5)Report on results unrelated to the effect of hepatic injury in rodents with hepatic fibrosis.

6) The data of evaluation indicators is incomplete (such as the measurement units of unmarked evaluation indicators).

Methods for data extraction

The two independently extracted relevant data. When any disagreement arises, it can be resolved through discussion or through third-party intervention until a consensus is reached. Outcome indicators must be presented in numerical form to ensure that key indicators, including their mean and standard deviation, can be directly extracted or indirectly calculated. Outcome measures were excluded when there were fewer than 3 articles on a single outcome measure. We will contact the study authors by email to seek to provide missing or additional data, etc. .

Data to be extracted: study design

1) Research subjects (experimental group, control group, and number of animals in each group).

2) Modeling methods.

3) Administration method and dosage (mg/kg).

4) Result indicators.

Data to be extracted: animal model

Species, strain, sex.

Data to be extracted: intervention of interest

The maximum dose and the longest time of curcumin and curcumin in the intervention of hepatic fibrosis model.

Data to be extracted: primary outcome(s)

hyaluronic acid (HA);continuous;ng/ml.

laminin (LN);continuous;ng/ml.

type III procollagen (PC III);continuous;ng/ml.

type IV collagen (IV-C);continuous;ng/ml.

Data to be extracted: secondary outcome(s)

alanine aminotransferase (ALT);continuous;U/L.

aspartate aminotransferase (AST);continuous;U/L.

alkaline phosphatase (ALP);continuous;U/L.

albumin (ALB);continuous; g/L.

ratio of albumin to globulin (A/G);continuous; %.

total protein (TP);continuous;g/L.

total bilirubin (TBIL);continuous;mg/dl.
superoxide dismutase (SOD);continuous;U/mg.
malondialdehyde (MDA);continuous;nmol/mg.
glutathione peroxidase (GSH-Px);continuous;U/mg.

Data to be extracted: other

Author, year of publication, drug use, method of modeling, number of models, method of administration, dosage of administration, detection index, detection index units and results

27. * Risk of bias and/or quality assessment.

State whether and how risk of bias and/or study quality will be assessed. Assessment tools specific for pre-clinical animal studies include SYRCLE's risk of bias tool and the CAMARADES checklist for study quality

No risk of bias and/or quality assessment planned
No

By use of SYRCLE's risk of bias tool
Yes

By use of SYRCLE's risk of bias tool adapted as follows:
No

By use of the CAMARADES checklist for study quality
No

By use of the CAMARADES checklist for study quality, adapted as follows:
No

Other criteria, namely
No

Method for risk of bias and/or quality assessment
The methodological quality of the included studies was expected to be assessed by using the SYRCLE bias risk tool. Each person extracts data independently, and any disagreements can be resolved through discussion or third-party intervention until a consensus is reached.

28. * Strategy for data synthesis. [1 change]

Planned approach
We will be Quantitative analysis to bring together terms and measures with common definitions into a single unified term. Conversion of results to uniform units of measure, including conversion of the mean or median and interquartile range of standard errors to mean and standard deviation. Meta-analyses were performed using RevMan 5.1 software if a sufficient number of studies were performed using similar methods (≥3) .

Effect measure
hyaluronic acid (HA);mean difference.
laminin (LN);mean difference.
type III procollagen (PC III);mean difference.
type IV collagen (IV-C);mean difference.
alanine aminotransferase (ALT);mean difference.
aspartate aminotransferase (AST);mean difference.
alkaline phosphatase (ALP);mean difference.
albumin (ALB);mean difference.
ratio of albumin to globulin (A/G);odds ratio.
total protein (TP);mean difference.
total bilirubin (TBIL);mean difference.
superoxide dismutase (SOD);mean difference.

malondialdehyde (MDA);mean difference.
glutathione peroxidase (GSH-Px);mean difference.

Effect models
When there was significant heterogeneity, we chose a random-effects model for meta-analysis; otherwise, we used a fixed-effects model.

Heterogeneity
Cochran's Q test and I^2 test were used to assess the presence and severity of heterogeneity. Heterogeneity was considered when $p < 0.1$ and I 2 > 50% .

Other
Not yet.

29. * Analysis of subgroups or subsets. [1 change]

Subgroup analyses
To investigate the effect of curcumin on hepatic fiber models in different animal species, we classified the included literature according to rat versus mouse when there were fewer than 3 relevant articles on rat or mouse outcome measures; The outcome measure will be dropped.

Sensitivity
Stata software to analyze the sensitivity of each group of data.

Publication bias
We evaluated publication bias using BEGG rank correlation and Egger linear regression in Stata 12.0. If there is publication bias, we will further use the cut-and-fill method to analyze whether this outcome indicator will be reversed.

30. * Review type.

Type of review

Animal model review	Yes
Experimental animal exposure review	Yes
Pre-clinical animal intervention review	No

31. Language.

Select each country individually to add it to the list below, use the bin icon to remove any added in error.

English

There is not an English language summary

32. * Country.

Select the country in which the review is being carried out from the drop down list. For multi-national collaborations select all the countries involved.

China

33. Other registration details.

List other places where the systematic review protocol is registered. The name of the organisation and any unique identification number assigned to the review by that organisation should be included.

34. Reference and/or URL for published protocol.

Give the citation and link for the published protocol, if there is one.

No I do not make this file publicly available until the review is complete

35. Dissemination plans.

Give brief details of plans for communicating essential messages from the review to the appropriate audiences.

No

36. * Keywords.

Give words or phrases that best describe the review. Separate keywords with a semicolon or new line.

Curcumin; hepatic fibrosis; rodents,; meta-analysis

37. Details of any existing review of the same topic by the same authors.

Give details of earlier versions of the systematic review if an update of an existing review is being registered, including full bibliographic reference if possible.

38. * Current review status.

Review status should be updated when the review is completed and when it is published.

Review_Ongoing

39. Any additional information.

Provide any further information the review team consider relevant to the registration of the review.

40. Details of final report/publication(s) or preprints if available.

This field should be left empty until details of the completed review are available OR you have a link to a preprint. Give the full citation for the preprint or final report or publication of the systematic review.
